# Clinical-histological characteristics and therapeutic management of primary cutaneous melanoma in elderly patients^☆^

**DOI:** 10.1016/j.abd.2024.07.017

**Published:** 2025-04-09

**Authors:** Juan-Manuel Morón-Ocaña, Isabel-María Coronel-Pérez, Ana-Isabel Lorente-Lavirgen, Carmen-Victoria Almeida-González, Amalia Pérez-Gil

**Affiliations:** a Department of Dermatology, Hospital Universitario Virgen de Valme, Sevilla, Spain; b Unit of Statistics and Research Methodology, Hospital Universitario Virgen de Valme, Sevilla, Spain

**Keywords:** Aged, Melanoma, Therapeutics

## Abstract

**Background:**

Life expectancy is rising in developed countries. The impact of age on melanoma characteristics is unclear, but it seems that melanomas in the elderly have distinct features affecting management and outcomes.

**Objectives:**

To compare clinical and histopathological melanoma characteristics and management in elderly and younger patients.

**Methods:**

A retrospective population-based study analyzed melanomas observed between 2007 and 2022 was made in the southern Seville health area (Spain). Patients were divided into two age groups: <65 and ≥65. Data were collected from clinical histories.

**Results:**

Among 431 primary cutaneous melanomas, 33% were in patients ≥65-years. Elderly patients had more head and neck melanomas (37.8% vs. 14.9%; p < 0.001), larger lesions (1.3 vs. 0.9 cm; p < 0.001), more ulcerated melanomas (17.8% vs. 8.8%; p < 0.012), and higher Breslow thickness (1.03 vs. 0.65 mm; p < 0.01) than younger patients. No differences were found in the number of mitoses or histopathological invasions. Stage 0 and more advanced stages (II/III/IV) were observed more frequently in ≥65-years (29.3% vs. 23% and 27.1% vs. 15.7%, p < 0.001 respectively). Fewer wide excisions (28.4% vs. 5.6%, p < 0.001), sentinel lymph node biopsy (17.6% vs. 2.4%, p < 0.001), and adjuvant therapy (11.9% vs. 2.1%, p < 0.001) were performed in patients ≥65-years.

**Study limitations:**

The study was retrospective, primarily covering the last 10-years, with older data missing. Key risk factors like the number of nevi and family history of melanoma were not collected.

**Conclusions:**

Melanomas in the elderly were diagnosed more frequently at initial and advanced stages despite having worse prognostic characteristics compared melanomas occurring in younger people.

## Introduction

As the population continues to age, the healthcare system will be faced with the prospect of caring for an increasing number of elderly individuals with diagnoses of cancer. In 2019, more than a fifth (20.3%) of the population of the European Union (EU) was 65-years of age or older. Projections indicate that the percentage of people aged 80 or over in the EU population will multiply by 2.5 between 2019 and 2100, from 5.8% to 14.6%.^1^

Although the incidence and mortality of cancer are generally higher in the older age groups, few studies have dealt primarily with elderly patients with melanoma. The incidence of melanoma and related mortality has been steadily increasing since 1970 in most developed countries.^2^ In the United States, more than 40% of melanomas are diagnosed in patients older than 65-years.^3^

In Spain, incidence rates are estimated to be 16 per 100,000 in 2022. By age, the data indicates that the group that will have a higher incidence is that of ≥65-years, with 44% of the cases; followed by the group of 45- to 64-years, with 39%; and, finally, the one from 0 to 44-years old, with 17%.^4^

Although the number of melanoma cases among the elderly (>65-years) is expected to increase owing to the aging population, the influence of age on the characteristics of and outcome of melanoma is unclear. In the international literature, classical factors associated with a poorer prognosis in older patients are nodular subtype, tumors with a higher Breslow thickness, a higher mitotic index, and more advanced stages at diagnosis.^5^, ^6^, ^7^, ^8^, ^9^

Cancers in the elderly population, including melanoma, have features that distinguish them from cancers in younger cohorts and potentially affect the management and outcome of these patients. The present study evaluated to what extent clinical and histologic characteristics of primary cutaneous melanomas and their therapeutic management differed in older patients (≥65) compared with younger ones (<65).

## Methods

### Study population

The study was performed with the people belonging to Valme University Hospital, which provides health care to a population of about 500,000 inhabitants in the southern area of Seville (Spain). The study included patients over 18-years old who were diagnosed with primary cutaneous melanoma in the last 15-years and are still being followed up in the Oncological Dermatology unit of Valme Hospital. Patients under 18-years and patients with mucosal, ocular, lymph node melanoma or unknown primary melanoma were excluded.

### Data collection

The study was approved by the ethics committee of Valme University Hospital in Seville (code 1765-N-22). Two groups were established in the study according to their age. Older patients were defined as individuals aged 65-years or older and were compared with those younger than 65-years (the younger group) for every study variable.

The following data were collected for each patient: age diagnosis of first/unique melanoma, sex, area of residence (urban vs. rural), sun exposition (occupational vs. recreational), sunburn (ever vs. never), use of UV cabins (ever vs. never) and phototype (I to VI).

The following data were collected for each melanoma: age diagnosis, anatomic location (head and neck, trunk, upper extremities, and lower extremities), lesion size (cm), histologic subtype (including superficial spreading melanoma [SSM], nodular melanoma, lentigo malignant melanoma [LMM], acral lentiginous melanoma, and other or unclassified subtypes), Breslow thickness (mm), ulceration (yes vs. no), mitosis (number), histological invasion (yes vs. no) and associated melanocytic lesion (yes vs. no).

Data of initial management was classified as excision biopsy (indicated done vs. indicated dismissed), definitive margin excision (indicated done vs. indicated dismissed), selective lymph node biopsy (SLNB) (indicated done vs indicated dismissed vs. no indicated), adjuvant therapies (indicated received vs. indicated dismissed vs no indicated). The initial stage was classified as 0, I, II and III/IV stages.

### Statistical analysis

Quantitative variables were described as means and standard deviations or with medians and quartiles in the case of asymmetric distributions. Qualitative variables were described as numbers and percentages.

Different hypothesis contrasts were applied depending on the variables at play. Comparisons between the older and younger groups were performed using the χ^2^ test, non-asymptotic Monte Carlo methods, Mann-Whitney *U*-test, Anova model, or the Kruskall-Wallis test, as appropriate; p < 0.05 was considered statistically significant. In cases of significance, 95% Confidence Intervals were calculated for the mean and prevalence estimates. Statistical analyses were performed using the statistical software IBM SPSS 28.0.

## Results

**Fig. 1:**
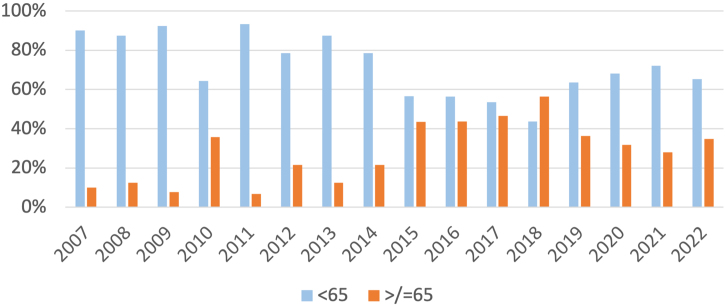
Evolution of melanoma incidence between 2007‒2022 in elderly people.

A total of 399 patients with primary cutaneous melanoma were included in the study. A total of 431 primary cutaneous melanomas were analyzed. Between 2007‒2022, 33% of patients with primary cutaneous melanoma were older than 65-years. The number of primary melanomas diagnosed in people over 65-years has been increasing from approximately 10% between 2007‒2009 to an average of 35% in the last years (Fig. 1).

### Patient characteristics

The median age of the first/unique primary melanoma in the older and younger groups was 74 (IQR = 69.75‒78.25) and 48 (IQR = 37‒55.5), respectively, with statistically significant differences (p < 0.001). Significant differences were also observed for sunburns (29.6% vs. 70.4% p < 0.001) and use of UV cabins (1% vs. 14.2% p < 0.001). No significant differences were observed for sex, area of residence, sun exposition and phototype (Table 1).

**Table 1:** Demographic characteristics of the patients at the time of diagnosis of their first melanoma.

Characteristic	Nº (%)			p
	Total	Age (years)		
	n = 399 (100%)	< 65	≥ 65	
		n = 265 (66.4%)	n = 134 (33.6%)	
**Age (first/unique melanoma)**				
Total	399 (100%)	265 (66.4%)	134 (33.6%)	< 0.001
Mean (± SD)	56 (±16.62)	75 (±6.35)	46 (±11.22)	
Median (RIQ)	56 (44‒70)	48 (37‒55.5)	74 (69.75‒78.25)	
**Sex**				
Total	399 (100%)	265 (66.4%)	134 (33.6%)	NS
Male	199 (49.9%)	134 (50.6%)	65 (48.5%)	
Female	200 (50.1%)	131 (49.4%)	69 (51.5%)	
**Area of residence**				
Total	398 (99.7%)	265 (66.6%)	133 (33.4%)	NS
Rural	179 (45%)	119 (44.9%)	60 (45.1%)	
Urban	219 (55%)	146 (55.1%)	73 (54.9%)	
**Sun exposition**				
Total	358 (89.7%)	252 (70.4%)	106 (29.6%)	NS
Occupational/chronic	118 (33%)	76 (30.2%)	42 (39.6%)	
Recreational	240 (67%)	176 (69.8%)	64 (60.4%)	
**Sun burns**				
Total	358 (89.7%)	252 (70.4%)	106 (29.6%)	<0.001
Ever	224 (62.6%)	177 (70.2%)	47 (44.3%)	
Never	134 (37.4%)	75 (29.8%)	59 (55.7%)	
**Use of UV cabins**				
Total	348 (87.2%)	247 (71%)	101 (29%)	<0.001
Ever	36 (10.3%)	35 (14.2%)	1 (1.0%)	
Never	312 (89.7%)	212 (85.9%)	100 (99%)	
**Phototype**				
Total	368 (92.2%)	255 (69.3%)	113 (30.7%)	NS
I	1 (0.3%)	1 (0.4%)	0 (0.0%)	
II	168 (45.7%)	120 (47.1%)	48 (42.5%)	
III	157 (42.7%)	104 (40.8%)	53 (46.9%)	
IV	41 (11.1%)	30 (11.8%)	11 (9.7%)	
V	1 (0.3%)	0 (0.0%)	1 (0.9%)	
VI	0 (0.0%)	0 (0.0%)	0 (0.0%)	

NS, Not Significative.

### Melanoma characteristics

The median age of the melanoma diagnosed in the older and younger groups was 74 (IQR = 70‒78) and 48 (IQR = 37.55‒75). Melanomas located on the head and neck were more frequent in people over 65-years (37.8% vs. 14.9%; p < 0.001). Although the most frequent histological subtype was the SSM, its frequency decreased considerably with respect to those under 65-years (43.4% vs. 74.7% p < 0001). There was an increase of LMM (35% vs. 8.7%, p < 0.001) and nodular melanoma (14% vs. 9.4%, p < 0.001) in elderly patients. Those older than 65-years had larger melanomas (median 1.3 cm (IQR = 1‒2) vs. median 0.9 cm (IQR = 0.6‒1.3), p < 0.001), higher Breslow thickness (median of 1.03 mm (IQR = 0.5‒3.1) vs. median of 0.65 (IQR = 0.48‒1.3), p < 0.01 and more ulcerated melanomas (17.8% vs. 8.8%, p < 0.012). There were no differences in the number of mitoses or the presence of invasion (lymphatic, vascular, neural). On the other hand, melanomas in people over 65-years were less frequently associated with previous melanocytic lesions (21.3% vs. 35.5%, p = 0.022). Patients under 65-years doubled the risk of presenting a melanocytic lesion prior to melanoma (OR = 2.03 with 95% CI [1.1‒3.7], p < 0.005), which could be multiplied by almost four (Table 2).

**Table 2:** Initial clinical and histological characteristics of melanoma according to age.

Characteristic	No. (%)			p
	Total	Age (years)		
	n = 431 (100%)	< 65	≥ 65	
		n = 288 (66.8%)	n = 143 (33.2%)	
**Age**				
Total	431 (100%)	288 (66.8%)	143 (33.2%)	<0.001
Mean (±SD)	55.66 (±16.45)	46,31 (±11.13)	74.51 (±6.20)	
Median (RIQ)	55 (44‒70)	48 (37‒55.75)	74 (70‒78)	
**Location**				
Total	431 (100%)	288 (66.8%)	143 (33.2%)	<0.001
Head and neck	97 (22.5%)	43 (14.9%)	54 (37.8%)	
Trunk	179 (41.5%)	140 (48.6%)	39 (27.3%)	
Uper limb	60 (13.9%)	39 (13.5%)	21 (14.7%)	
Lower limb	95 (22%)	66 (22.9%)	29 (20.3%)	
**Size (cm)**				
Total	383 (88.9%)	254 (66.3%)	129 (33.7%)	<0.001
Mean (±SD)	1.6 (2.54)	1.25 (±1.8)	2.53 (±3.5)	
Median (RIQ)	0.75 (0.5‒1.6)	0.65 (0.48‒1.3)	1.03 (0.5‒3.1)	
**Subtipo histológico**				
Total	431 (100%)	288 (66.8%)	143 (33.2%)	<0.001
SSM	277 (64.3%)	215 (74.7%)	62 (43.4%)	
Nodular	47 (10.9%)	27 (9.4%)	20 (14%)	
LMM	75 (17.4%)	25 (8.7%)	50 (35%)	
ALM	19 (4.4%)	11 (3.85)	8 (5.6%)	
Other	13 (3%)	10 (3.5%)	3 (2.1%)	
**Breslow thickness (mm)**				
Total	429 (99.5%)	288 (67.1%)	141 (32.9%)	<0.01
Mean (±SD)	1.65 (±2.54)	1.25 (±1.83)	2.53 (±3.5)	
Median (RIQ)	0.75 (0.5‒1.6)	0.65 (0.48‒1.3)	1.03 (0.5‒3.1)	
**Ulceration**				
Total	378 (87.7%)	249 (65.9%)	129 (34.1%)	0.012
Yes	45 (11.9%)	22 (8.8%)	23 (17.8%)	
No	333 (88.1%)	227 (91.2%)	106 (82.2%)	
**Mitosis (number)**				
Total	351 (81.4%)	227 (64.7%)	124 (35.3%)	NS
Mean (±SD)	1.82 (±4.03)	1.68 (±4.15)	2.07 (±3.81)	
Median (RIQ)	0 (0‒2)	0 (0‒1.)	1 (0‒2.75)	
**Histological invasion (lymphatic/perineural/vascular)**				
Total	319 (74.0%)	208 (65.2%)	111 (34.8%)	NS
Yes	16 (5%)	7 (3.4%)	9 (8.1%)	
No	303 (95%)	201 (96.6%)	102 (91.9%)	
**Associated melanocytic lesion**				
Total	249 (57.8%)	155 (62.2%)	94 (37.8%)	0.022
Yes	75 (30.1%)	55 (35.5%)	20 (21.3%)	
No	174 (69.9%)	100 (64.5%)	74 (78.7%)	

NS, Not Significative; SSM, Superficial Spreading Melanoma; LMM, Lentigo Malignant Melanoma; ALM, Acral Lentiginous Melanoma.

### Initial tumor stage and initial management of primary cutaneous melanoma

Stage 0 and more advanced stages (II/III/IV) were observed more frequently in those over 65-years (29.3% vs. 23%, p < 0.001 and 27.1% vs. 15.7%, p < 0.001 respectively), while the I stage prevailed in those under 65 (61.3% vs. 43.6%, p < 0.001) (Table 3). Regarding therapeutic management, there were no differences in the type of primary melanoma resection (NS). However, fewer wide excisions (28.4% vs. 5.6%, p < 0.001) and fewer SLNB (17.6% vs. 2.4%, p < 0.001) were performed in those over 65-years of age although they were indicated. Furthermore, adjuvant therapy was started less frequently in older patients (11.9% vs. 2.1%, p < 0.001).

**Table 3:** Stage and initial management of cutaneous melanoma according to age.

Characteristic	No. (%)			p
	Total	Age (years)		
	n = 431 (100%)	< 65	≥ 65	
		n = 288 (66.8%)	n = 143 (33.2%)	
**Initial staging**				
Total	427 (98.4%)	287 (67.2%)	140 (32.8%)	<0.001
0 (in situ)	107 (25.1%)	66 (23%)	41 (29.3%)	
I	237 (55.5%)	176 (61.3%)	61 (43.6%)	
II	43 (10.1%)	20 (7%)	23 (16.4%)	
III/IV	40 (9.3%)	25 (8.7%)	15 (10.7%)	
**Excision**				
Total	424 (98.4%)	281 (66.3%)	143 (33.7%)	NS
Indicated done	422 (99.5%)	281 (100%)	141 (98.6%)	
Complete	396 (93.4%)	268 (95.4%)	128 (89.5%)	
Incomplete	26 (6.1%)	13 (4.6%)	13 (9.09%)	
Indicated dismissed	2 (0.5%)	0 (0%)	2 (1.4%)	
**Wide excision**				
Total	429 (99.5%)	288 (67.1%)	141 (32.9%)	<0.001
Indicated done	373 (86.9%)	272 (94.4%)	101 (71.6%)	
Positive	15 (3.5%)	7 (2.4%)	8 (5.7%)	
Negative	358 (83.4%)	265 (92%)	93 (66%)	
Indicated dismissed	56 (13.1%)	16 (5.6%)	40 (28.4%)	
**SLNB**				
Total	430 (99.8%)	288 (67%)	142 (33%)	<0.001
Indicated done	134 (31.2%)	95 (33%)	39 (27.5%)	
Positive	32 (7.4%)	21 (7.3%)	11 (7.7%)	
Negative	102 (23.7%)	74 (25.7%)	28 (19.7%)	
Indicated dismissed	32 (7.4%)	7 (2.4%)	25 (17.6%)	
No indicated	264 (61.4%)	186 (64.6%)	78 (54.9%)	
**Adjuvant therapy**				
Total	431 (100%)	288 (66.8%)	143 (33.2%)	<0.001
Indicated	61 (14.1%)	33 (11.5%)	28 (19.6%)	
Received	38 (8.8%)	27 (9.4%)	11 (7.7%)	
Dismissed	23 (5.3%)	6 (2.1%)	17 (11.9%)	
No indicated	370 (85.8%)	255 (88.5%)	115 (80.4%)	

NS, Not Significative; SLNB, Selective Lymph Node Biopsy.

## Discussion

The present results confirm and extend those of previous reports on the presentation and characteristics of melanoma in the elderly. In accordance with the previous series,^5^, ^10^, ^11^ the authors observed that the head and neck were the most frequent location in older patients (37.8%). Previous authors have demonstrated that the head and neck location of melanoma increases with age and becomes the most common site after age 70-years.^12^, ^13^ It was suggested that this high incidence of head and neck melanoma might be attributable to cumulative photodamage, resulting notably in numerous LMM.

As in previous studies, the most relevant difference in terms of baseline prognostic characteristics between older and younger patients was a higher Breslow thickness in the older group.^5^, ^10^, ^11^, ^12^, ^13^ Other classic poor prognostic characteristics such as larger clinical size and ulceration were also observed in the elderly group as in previous studies.^5^, ^10^, ^11^, ^12^, ^13^

While SSM is the most frequent histological subtype in younger age groups,^8^ in the patients ≥65-years, there is disproportionately an excess of both lentigo malignant (35%) and nodular melanomas (14%), with fewer superficial spreading melanomas (43.4%). In most series, (NM ranks second in frequency^5^, ^7^; However, in this study, the percentage of lentigo maligna/melanoma (LM/M) is in second position, which is likely significantly related to chronic sun exposure in the studied region. The excess of LMM seen in this study supports the association between LMM, advanced age and chronically sun-exposed skin.^14^

Stage 0 and more advanced stages (II/III/IV) were observed more frequently in those over 65-years. Among factors that could explain this difference, the frequency of melanomas in situ and the nodular histologic subtype may play an important role. In the present study, melanoma in situ and nodular melanoma occurred 1.3 and 1.5, respectively, as frequently in the older group as in the younger one. The frequency of nodular melanomas, which are highly malignant, and progress rapidly may also explain the higher IV stage in older patients. In addition to nodular melanoma, poor prognosis in the older could be explained because of a delayed diagnosis as a consequence of location in scarcely visible areas (scalp and back), absence of a partner for home examination, poor vision, ignorance of clinical changes, and/or confusion between melanoma and seborrheic keratoses.^12^

Following initial diagnosis, the management of melanoma also appears to be a challenging issue. Chang et al.^15^ emphasized that older patients should be treated according to the characteristics and prognostic factors of their tumors and not according to their more advanced age. In contrast to this statement, the authors observed that 28.4% of elderly people, compared with 5.6% of younger ones, had no wide excision.

Among patients typically eligible for SLNB, 17.3% of the older group vs. 2.4% of the younger one underwent this procedure just for the age or comorbidities. This may partly be the result of the limited practical value of SLNB in older patients for whom adjuvant therapies are not an option. However, the role of SLNB in older patients is contentious given their limited life expectancy and the presence of other competing causes of mortality; however, it is recommended that the decision to employ or go SLNB be evaluated on a case-by-case basis.^11^

Another major difference between older and younger patients was the rate of adjuvant therapy proposed and completed. The 11.9% of older patients compared with 2.1% of younger ones eligible for adjuvant therapy did not undergo the full course of therapy. Many older patients have poor health, making it difficult to prescribe adjuvant therapies. In addition, older patients may be reluctant to accept a treatment with significant adverse effects and little benefit.

There are some limitations to this study. It was a retrospective study and what the authors observed in the area may not be generalized to other territories. Most of the patients of this study have been collected in the last 10-years. Although many of the patients diagnosed before 2012 were discharged and the authors do not have their data, no significant differences were observed between these years for the characteristics of patients, melanomas and main features of management except for an increase in the number of primary cutaneous melanoma diagnosis in the last years. Other factors such as number of nevi, family history of melanoma, and history of nonmelanoma skin cancer or noncutaneous cancer were not collected. Further studies are required to compare such risk factors and characteristics between older and patients younger.

## Conclusions

In summary, the number of cases of melanoma in people over 65 has been increasing in the last decade. Compared with the younger group, older patients had primary cutaneous melanomas more frequent in the head and neck with higher Breslow thickness, larger clinical size, and ulceration. Melanomas were diagnosed more frequently in initial (in situ) and advanced (II, III, IV) stages and they have less association with previous lesions. In addition, more interventions and oncological treatments are ruled out solely because of the age despite the fact there are no contraindications.

Stage at diagnosis remains the most important difference between younger and older patients. Further public health campaigns regarding melanoma should focus on access of elderly people to early diagnosis and excision with appropriate margins. An assessment of the patient's comorbidities anticipated life expectancy, frailty, and ability to withstand the proposed treatment should be considered in planning the management of these patients.

## Financial support

None declared.

## Authors’ contributions

Juan-Manuel Morón-Ocaña: Preparation and writing of the manuscript; critical literature review.

Isabel-María Coronel-Pérez: Approval of the final version of the manuscript; manuscript critical review.

Ana- Isabel Lorente-Lavirgen: Approval of the final version of the manuscript; manuscript critical review,

Carmen-Victoria Almeida-González: Statistical analysis; study conception and planning.

Amalia pérez-gil: approval of the final version of the manuscript; manuscript critical review.

## Conflicts of interest

None of the authors have any conflict of interests to declare.
